# Adsorption Mechanisms of Dodecylbenzene Sulfonic Acid by Corn Straw and Poplar Leaf Biochars

**DOI:** 10.3390/ma10101119

**Published:** 2017-09-22

**Authors:** Nan Zhao, Xixiang Yang, Jing Zhang, Ling Zhu, Yizhong Lv

**Affiliations:** 1 College of Resources and Environmental Sciences, China Agricultural University, Beijing 100193, China; zhaonan8@mail.sysu.edu.cn (N.Z.); zhuling@cau.edu.cn (L.Z.); 2 Guangdong Provincial Key Lab of Environmental Pollution Control and Remediation Technology, School of Environmental Science and Engineering, Sun Yat-sen University, Guangzhou 510275, China; 3 Department of Applied Chemistry, Graduate School of Engineering, Kyushu University, 744 Motooka, Nishi-ku, Fukuoka 819-0395, Japan; qiqiang2010@yeah.net; 4 Department of Environmental Nano-Materials, Research Center for Eco-Environmental Sciences, Chinese Academy of Sciences, Beijing 100085, China; jingzhang@rcees.ac.cn

**Keywords:** corn straw, poplar leaf, biochar, structural characteristics, adsorption mechanism

## Abstract

Biochar is an eco-friendly, renewable, and cost-effective material that can be used as an adsorbent for the remediation of contaminated environments. In this paper, two types of biochar were prepared through corn straw and poplar leaf pyrolysis at 300 °C and 700 °C (C300, C700, P300, P700). Brunaer–Emmett–Teller N_2_ surface area, scanning electron microscope, elemental analysis, and infrared spectra were used to characterize their structures. These biochars were then used as adsorbents for the adsorption of dodecylbenzene sulfonic acid (DBSA). The microscopic adsorption mechanisms were studied by using infrared spectra, ^13^C-nuclear magnetic resonance spectra, and electron spin resonance spectra. The surface area and pore volume of C700 (375.89 m^2^/g and 0.2302 cm^3^/g) were the highest among all samples. Elemental analysis results showed that corn straw biochars had a higher aromaticity and carbon to nitrogen (C/N) ratio than the poplar leaf biochars. High temperature caused the increase of carbon content and the decrease of oxygen content, which also gave the biochars a higher adsorption rate. Pseudo-second order kinetic provided a better fit with the experimental data. Adsorption isotherm experiments showed that the adsorption isotherm of C300 fit the linear model. For other biochars, the adsorption isotherms fitted Langmuir model. Biochars with high temperatures exhibited enhanced adsorption capacity compared with ones at low temperatures. The *q_max_* values of biochars to DBSA followed the order of P700 > C700 > P300. The adsorption mechanisms were complex, including partition, anion exchange, the formation of H bonds, covalent bonds, and charge transfer. The adsorption by covalent bonding might be the key mechanism determining the adsorption capacity of P700.

## 1. Introduction

Linear alkylbenzene sulfonates (LAS) are popularly used in detergents and cleaning products. They can cause environmental hazards after entering into surface waters by household and industrial sewage [1]. Some techniques have been applied for the removal of surfactants from water or soils. Adsorption is a superior method for water treatment or soil remediation due to its low cost, easy operation, and high efficiency. Mineral oxides, clays, activated carbon, and polymers can be used as the adsorbents for the adsorption of surfactants [2], but the sorption capacity of sodium dodecyl sulfate on Fluvisol soil was very low [3].

Renewable bioenergy technologies can convert biomass feedstock into biochar, accompanied with the production of bio-gas and bio-oil [4,5]. Biochar is a carbonaceous material created through thermal pyrolysis of biomass without oxygen condition. The yield of biochar will decrease in the pyrolysis process, due to the dehydration and thermal degradation of cellulose and lignin structures [6]. The type of feedstock and pyrolysis temperatures can also influence the physical, chemical and adsorption properties of biochars. Wheat straw biochar had higher carbon and nitrogen contents and lower ash materials, as compared with corn straw biochar [7]. Pine sawdust biochar also had a higher adsorption capacity than that of green waste and corncob biochars [8]. Higher pyrolysis temperature could cause the increase of C content and the adsorption affinity of organic contaminants on biochars. At the same time, H, O, N and S contents decreased [9,10]. Biochars have strong adsorption capacities for contaminants, such as carbaryl [11], naphthalene [9], fluoroquinolone antibiotics [12], hexavalent chromium [5], and so on. There is very little information on the adsorption properties of LAS on biochars.

Westall et al. had studied the sorption of LAS on the sediment materials, and the results showed that Freundlich and virial equations fitted the isotherms well [1]. The sorption mechanisms included hydrophobic interaction, specific chemical interaction, or electrostatic interaction. Sodium dodecyl sulfate could also be adsorbed by hydrophobic interaction with organic matter of the soil and by ligand exchange or electrostatic attraction with kaolinite [13]. Sodium dodecylbenzene sulfonate (SDBS) is an anionic surfactant that is used extensively worldwide. Many papers had reported its adsorption behaviors and adsorption mechanisms. It could be adsorbed onto the organophilic bentonites or activated carbon by the monolayer adsorption with a relatively fast process [14,15]. However, the adsorption of SDBS on kaolinite was a two-stage process with an apparent multilayer formation [16].

Dodecylbenzene sulfonic acid (DBSA) is the acid form of SDBS. DBSA could be adsorbed on humic acids (HAs) by H bonds, hydrophobic interactions, and electron donor-acceptor mechanisms. Alluvial soil HA had a higher sorption capacity than that of Mollisol soil HA [17]. The adsorption behaviors of DBSA on biochars have been seldom reported. Corn straw and poplar leaf are commonly seen in China, and they are attractive feedstocks for biochar production. Therefore, we chose corn straw and poplar leaf as the biomass feedstocks to produce biochars in this study. The objective of the study was to obtain a deeper investigation of the influence of the pyrolysis temperature and biomass feedstocks on the physicochemical properties of biochars, and the adsorption behaviors of DBSA on different types of biochars. Further, infrared (IR) spectra, ^13^C-nuclear magnetic resonance (NMR) spectra and electron spin resonance (ESR) spectra were applied to study the adsorption mechanisms. The application of ESR provides a new insight into the adsorption mechanism of DBSA on biochars.

## 2. Materials and Methods

### 2.1. Materials

Dodecylbenzene sulfonic acid (95%) was purchased from TCI (TCI, Shanghai, China). Its critical micelle concentration (CMC) is 0.4134 g/L [18]. The biomasses of corn straw and poplar leaf were collected from Inner Mongolia and Beijing, China. Before the pyrolysis, corn straw and poplar leaf were washed to remove the ash. Then, the dried biomass feedstocks were put into stainless steel reactors (cylindrically shaped with a height of 10.5 cm and an inner diameter of 7.5 cm) and pyrolyzed in a Sx2-4-10 digital laboratory muffle furnace (Zhonghuan Lab Furnae Co., Ltd., Tianjin, China) at 300 °C and 700 °C for two hours with a heating rate of 10 °C/min. These products were mixed with 1 M HCl and 3 M HF to remove the ash materials. After washing with deionized water, the corn straw biochar and poplar leaf biochar were crushed and sieved to 0.25 mm. The obtained biochars were referred to as C300, C700, P300 and P700. HCl and HF (Sinopharm Chemical Reagent Co., Ltd., Shanghai, China) were all of analytical grade.

### 2.2. Test Method

#### 2.2.1. Biochar Characterization

Scanning electron microscope (SEM) images of the biochars were recorded by a Hitachi S-4800 scanning microscope (Hitachi, Tokyo, Japan). The Brunauer–Emmett–Teller (BET) nitrogen specific surface areas of biochars were determined by N_2_ adsorption–desorption at 77 K with a Tristrar II 3020 surface area analyzer (Micromeritics, Norcross, GA, USA). The contents of C, N, and H were determined in a Vario EL III elemental analyzer (Elementar, GmbH, Hanau, Germany). The O content was calculated based on the equation: O% = 100% − C% − H% − N% − ash content. The micro IR spectra were recorded in the range of 4000–600 cm^−1^ with a resolution of 4 cm^−1^ using a Nicolet Nexus-470 FTIR spectrometer (Thermo Nicolet, San Diego, CA, USA). The solid-state ^13^C-NMR spectra were acquired on a Bruker DSX2300 spectrometer (Bruker, Faellanden, Switzerland) operated at 100.37 MHz. The sample was packed in a 4 mm rotor with a contact time of 3000 μs. The scan times were more than 20,000. ESR spectra were undertaken using a Bruker ESP300 spectrometer (Bruker BioSpin, Billerica, MA, USA) and operated at 100 kHz modulation and 9.75 GHz microwave frequency.

#### 2.2.2. Batch Adsorption Experiments

The adsorption kinetics of DBSA at a constant concentration (120 mg/L, 20 mL) onto 60 mg biochars were examined at different sampling times (0, 1, 2, 5, 10, 16, 24, 36, 48, 60, and 72 h). The temperature was 25 ± 2 °C. After shaking at the speed of 130 rpm, the solution was centrifuged at 5000 rpm for 12 min, and filtered through a 0.22 μm membrane. The concentration of DBSA was measured at 223 nm using the UV757 spectrophotometer (Shanghai Accurate Scientific Instrument Co., Ltd., Shanghai, China). Each point containing the blank and control samples was conducted in triplicate.

Different concentrations of DBSA solution (15 mg/L–360 mg/L, 20 mL) were mixed with 60 mg biochars in a 50 mL flask and shaken for 48 h to reach adsorption equilibrium for obtaining the adsorption isotherms. Then the mixtures were treated as described above.

#### 2.2.3. Data Analysis

The amount of DBSA adsorbed was calculated by the following equation:

*q_e_* = *V*(*C* − *C_e_*)/*m* (1)

*R*% = (*C* − *C_e_*/*C*) × 100% (2)

where *V* is the volume of the solution (mL), *C* and *C_e_* are the initial and equilibrium concentrations (mg/L), *m* is the weight of the biochars (g), and *R*% represents the removal efficiency.

The relationships between the adsorption rate constants and the chemical compositions of biochars were examined using the Pearson correlation coefficient.

## 3. Results and Discussion

### 3.1. Characteristics of Biochars

**Figure 1:**
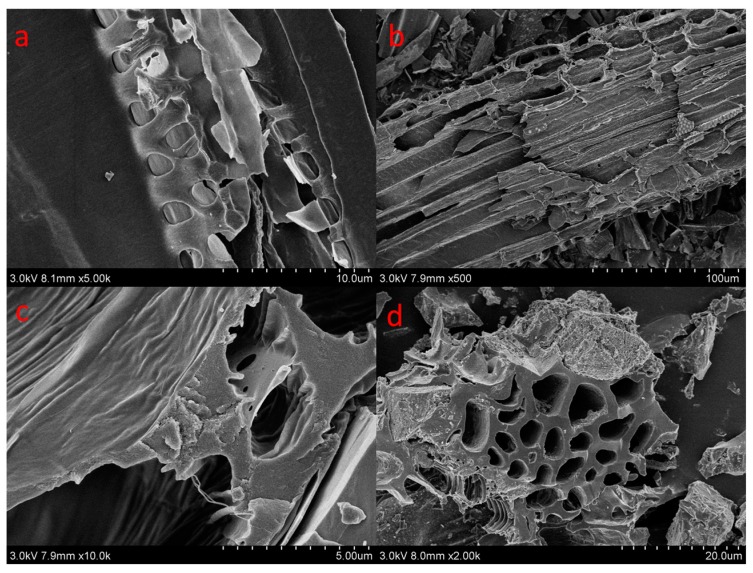
Scanning electron microscope images of C300 (**a**); C700 (**b**); P300 (**c**); and P700 (**d**).

**Figure 2:**
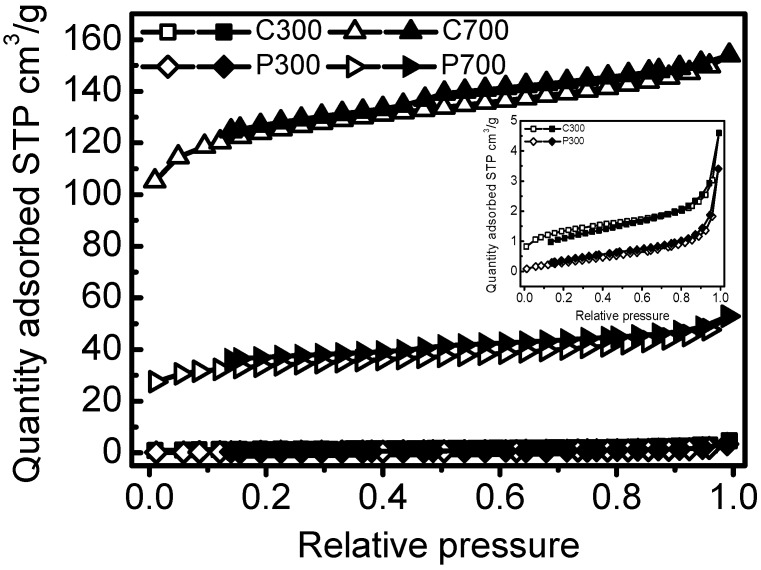
Adsorption and desorption isotherms for biochars. The hollow signs are for the adsorption branch, and the compact signs are for the desorption branch.

C700 and P700 samples exhibited larger surface areas (375.89 m^2^/g and 105.88 m^2^/g) and pore volumes (0.2302 cm^3^/g and 0.0721 cm^3^/g) than that of C300 and P300. Corn straw biochars also had higher surface areas and pore volumes by comparing with that of poplar leaf biochars. The average pore sizes of the biochars ranged from 3.98–12.27 nm (Table 1). The SEM micrographs of the biochars were demonstrated in Figure 1. C300 and P300 samples had fewer pores and more smooth appearances. At low temperature, amorphous carbons were the main carbons, and pores were not well developed. C700 and P700 presented uniform tunneling macropore structures in Figure 1b,d. C700 also had some smaller pore structures inside the tunneling macropores. Due to the high temperature, the tunneling structures had been crushed. The adsorption and desorption isotherms for biochars are shown in Figure 2. The similar isotherms of C700 and P700 suggested that the adsorption of N_2_ on biochars may follow similar kinetics [19] because of their similar pore structures. For C300 as well as for P300, the isotherms are characteristic of non-porous materials or materials with large macropores, indicating no porous structure. The elemental compositions and atom ratios of biochars are shown in Table 2. The C contents and C/N ratios of corn straw biochars were higher than those of poplar leaf biochars. The low ratios of H/C (0.21 and 0.38) indicated the high aromatization of biochars [20], because some of the amorphous carbon would be transformed into aromatic carbon at 700 °C [21]. The C contents, ash materials, and C/N ratios of the biochars were also greater at a higher pyrolyzing temperature. O/C was indicative of surface hydrophilicity, and (O + N)/C was recognized as the indice of polarity [22,23]. H, N, O, O/C, and (O + N)/C decreased with the pyrolyzing temperatures, due to the dehydration, decarboxylation, and decarbonylation in the pyrolysis process [24]. The distinct surface and structure characteristics of biochars, regulated by the biomass feedstock and pyrolytic temperatures, could significantly influence the adsorption of DBSA on biochars.

### 3.2. Adsorption Kinetics and Isotherm

**Figure 3:**
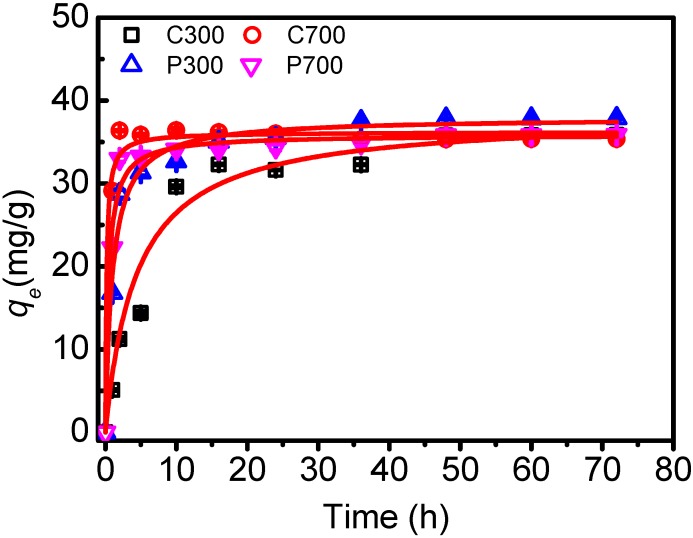
Adsorption dynamics for dodecylbenzene sulfonic acid (DBSA) on crop straw and poplar leaf biochars. The bars are the standard errors. The lines represent pseudo-second order kinetic fitting.

In Figure 3, results from studying the adsorption kinetics of DBSA on biochars are shown. The results indicated that the adsorption of DBSA on C700, P300, and P700 was relatively slow. However, it was faster than the adsorption of DBSA on soils and Mollisol soil HA [17]. Within 5 h of solution/biochar contact, 89.67%, 78.34%, and 83.09% of the initial DBSA amount were removed by C700, P300, and P700, respectively. After 10 h, the adsorption of DBSA almost reached its equilibrium with 91.05%, 81.69%, and 85.28% removal efficiencies. For C300, the adsorption seemed to approach equilibrium in 48 h, with a removal efficiency of 89.50%. Kinetic equation parameters for the adsorption of DBSA on biochars are listed in Table 3. Pseudo-second order kinetic was more suitable in describing the adsorption kinetics of DBSA on biochars (R^2^, 0.992–0.999). It was confirmed that chemical adsorption was the rate-limiting mechanism [22]. From the adsorption rate constant, it appears that DBSA adsorbed more quickly to the biochars prepared at high temperature than to the biochars made at low temperature.

**Figure 4:**
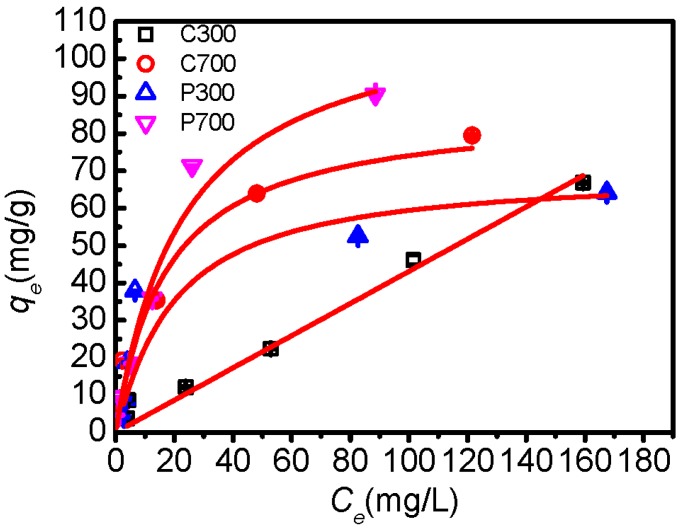
Adsorption isotherms for DBSA on crop straw and poplar leaf biochars. The bars are the standard errors. The lines represent linear and Langmuir equations fitting.

The adsorption isotherms of DBSA on biochars were demonstrated in Figure 4. DBSA adsorption capacity on biochars increased with the increasing pyrolysis temperature. Linear, Freundlich, and Langmuir models were applied to fit the isotherms, and the parameters of the isotherm equations were listed in Table 4. The linear model fit well for the adsorption of DBSA on C300, which indicated that the DBSA was partitioning into the non-carbonized organic matter of C300 [9]. The adsorption of DBSA on C700, P300, and P700 were best fit to the Langmuir isotherm under the concentration range studied, which confirmed that the adsorption occurred on a homogenous surface by monolayer adsorption [28]. It also indicated that a strong adsorption affinity existed between biochars and DBSA, and it was the anion exchange interaction that made the sulfonate ion adsorbed onto poplar leaf biochars and C700 [29]. Interestingly, the surface area of C700 was the biggest among four samples (Table 1), approximately 3.6 times that of P700 (Table 1), but *q_max_* of DBSA on P700 (155.9 mg/g) was the highest. Furthermore, P300 had the highest average pore size (12.27 nm), but the lowest *q_max_*. Therefore, surface area and average pore size were not the factor governing the adsorption capacity. This suggests that P700 has great potential in pollution control of surfactants.

In Table 5, the *K*_2_ values were analyzed as a function of some of the characteristics of the biochars (Table 1, Table 2, Table 6 and Table 7). A strong positive relationship was observed between the values of these adsorption rate constants and surface area, pore volume, and organic free radical concentrations of biochars. At the same time, *K*_2_ was negatively related to the content of O and (O + N)/C. The statistical analyses reported here have suggested the involvement of these structural parameters in the adsorption process.

### 3.3. Adsorption Mechanisms 

**Figure 5:**
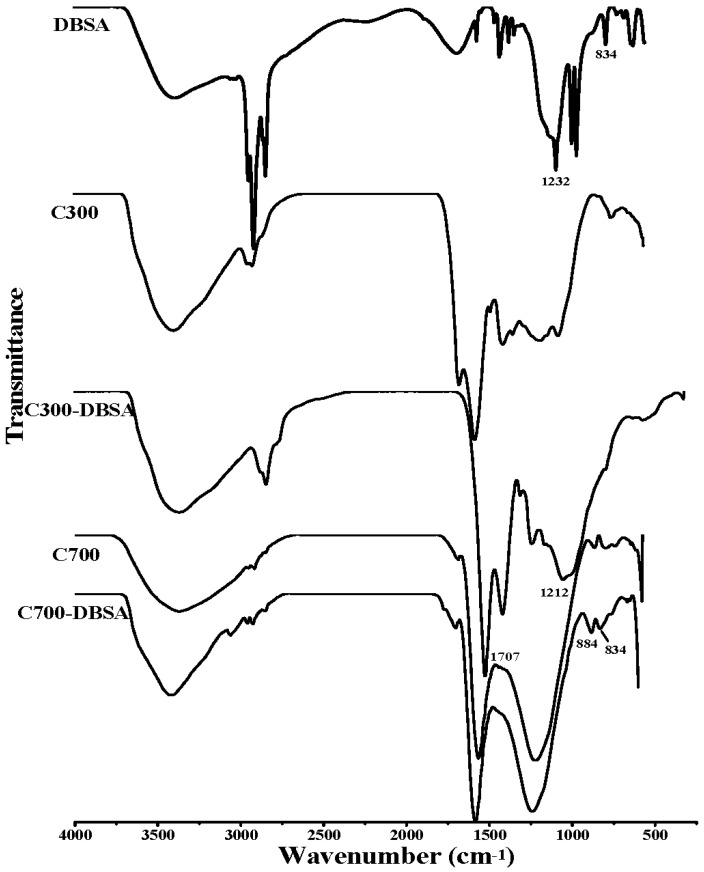
Infrared (IR) spectra of DBSA, crop straw biochars and their interaction complexes.

**Figure 6:**
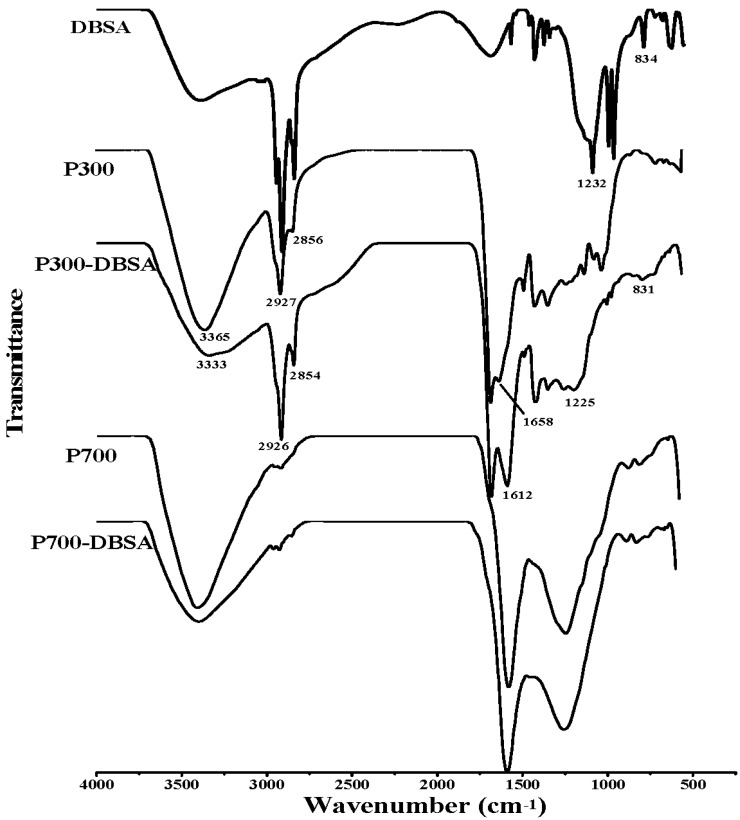
IR spectra of DBSA, poplar leaf biochars, and their interaction complexes.

As can be seen from the Fourier transform infrared (FTIR) spectra in Figure 5 and Figure 6, the broad band at 3365 cm^−1^ was ascribed to the O-H stretching vibration of hydroxyl functional groups [30]. The peaks at 2927 cm^−1^ and 2856 cm^−1^ were attributed to symmetric C-H stretching of alkyl groups [31]. P300 contained more alkyl groups than the other biochars, due to the stronger adsorption at 2927 cm^−1^. The bands at 1707 cm^−1^ and 1612 cm^−1^ were assigned to C=O vibrations [32]. The band at 1212 cm^−1^ was indicative of C-O stretching vibration in phenol and alcohol [33]. The peaks in 831–884 cm^−1^ corresponded to an aromatic C-H stretching vibration [34]. As can be seen from the FTIR results of C300, C700, P300, and P700, higher pyrolysis temperature caused the decrease of the aliphatic C-H groups, and the disappearance of the peaks at 1707 cm^−1^. These results were consistent with the elemental analysis results of lower ratios of H/C and O/C. The obvious changes of the structural characters might be the reason for the different adsorption sites and adsorption mechanism. The FTIR spectra of biochars after the adsorption of DBSA are also presented in Figure 5 and Figure 6. In Figure 5, the adsorption intensities at 1707 cm^−1^ and 1212 cm^−1^ in the FTIR spectrum of C300-DBSA became higher than that of the pristine C300 after the adsorption, which indicated that the adsorption took place in C=O and C-O groups, and H bond formed. H bond may be formed between the H atom in hydroxyl functional groups of phenol or carboxyl, and the O atom of DBSA. The presence of aromatic C-H groups at 884 cm^−1^ in the C700-DBSA sample confirmed that the aromatic C-H groups of DBSA were adsorbed on C700.

In Figure 6, after the adsorption of DBSA on P300, the band at 3365 cm^−1^ shifted to the lower frequency 3333 cm^−1^, which suggested the strong interaction formed through the H bond [35]. An increase in the intensity of the band at 1225 cm^−1^ could also confirm the formation of a H bond [33]. The peaks at 2926 cm^−1^and 2854 cm^−1^ became stronger, which indicated that the aliphatic C-H groups of DBSA were adsorbed on P300 [2]. The shape of the adsorbing peak of 1612 cm^−1^ became wider and the peak intensity increased, which could be ascribed to the adsorption on C=O groups. The appearance of an additional band at 831 cm^−1^ was the consequence of the interaction of aromatic C-H groups of DBSA with P300. The FTIR spectra of P700-DBSA showed no significant change in comparison to P700, which indicated that the adsorption of DBSA on P700 did not alter the structure.

**Figure 7:**
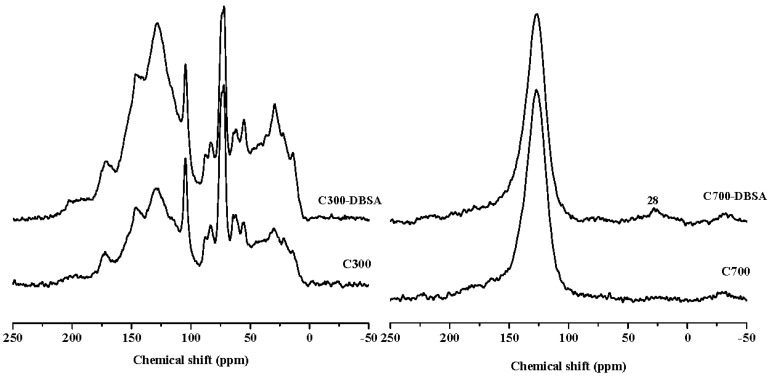
^13^C-NMR spectra of crop straw biochars, and their interaction complexes.

**Figure 8:**
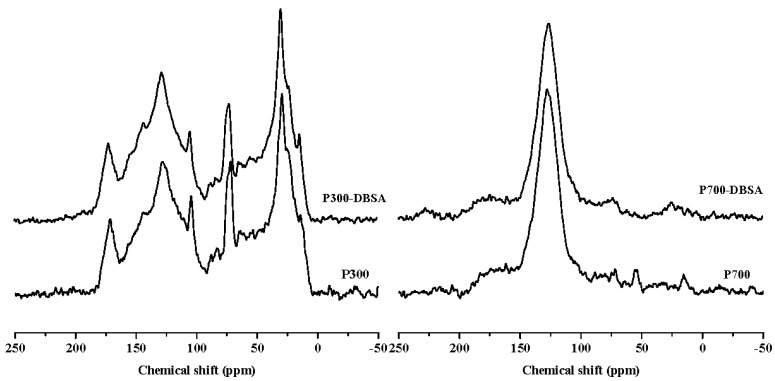
^13^C-NMR spectra of poplar leaf biochars and their interaction complexes.

Figure 7 and Figure 8 are the ^13^C-NMR spectra of biochars and biochars–DBSA complexes, respectively. The structural carbon distribution of the biochars and their complexes was calculated according to the ^13^C-NMR spectra, and is shown in Table 6. With the increase of the temperature, the contents of aliphatic carbon, heteroaliphatic carbon, and acetal carbon all decreased. At the same time, the contents of aromatic carbon, carboxyl carbon, and carbonyl carbon increased. Poplar leaf biochars had higher contents of aliphatic carbon and carboxyl carbon than corn straw biochars.

For C300, the content of aromatic carbon changed the most, from 35.98% before the adsorption to 42.22% after the adsorption. The change rate was the biggest (17.34%), which suggested that adsorption took place on aromatic carbon. The change rate of the aliphatic carbon of C700 was up to 4190%, and the presence of the long chain polymethylene carbon (28 ppm) implied that adsorption happened on long chain polymethylene carbon [36]. The adsorption mainly happened on carbonyl carbons of poplar leaf biochars due to the biggest change ratios of 78.13% and 77.89% for P300 and P700, respectively. After the adsorption of DBSA, the hydrophobicity of C700, Y300, and Y700 hardly changed.

Organic free radicals in humic acid or biochars have attracted considerable attention because of their potential for the adsorption and degradation of organic contaminants [37,38]. The *g* values observed for the biochars–DBSA complexes were the same as the *g* values of original biochars, thus indicating that no additional free radical species were generated [39]. Organic free radical concentrations increased in the interaction products of biochars and DBSA, relative to the original biochars, in the order: P700 > P300 > C300. These results could be the reason for the formation of charge-transfer between the electron donating carbonyl groups of biochars and the electron acceptor benzene ring of DBSA [40]. The enlargement of the line width in the interaction product of P700-DBSA was the consequence of the formation of covalent bonds between DBSA and P700 [39]. The covalent bonds might play a more important role in making the adsorption capacity of P700 higher than that of other biochars.

## 4. Conclusions

In this study, biochars from crop straw and poplar leaf have been prepared at 300 °C and 700 °C, respectively. The differences within the pyrolysis temperatures resulted in noticeable changes in the surface morphologies and structural characteristics of biochars derived from different biomass feedstocks. The adsorption kinetics of crop straw and poplar leaf biochars obeyed the pseudo-second order rate model. The study also investigated the adsorption abilities and adsorption mechanisms of DBSA on two different biochars. Isotherm analysis showed that the linear model fit well with the experiment data of C300, and the Langmuir model fit well with the data of C700, P300, and P700. The adsorption mechanisms of DBSA on crop straw biochars included partition, H bonds, and charge-transfer bonds. The adsorption sites were C=O, C-O, aromatic C, aromatic C-H groups, and long chain polymethylene carbon. IR spectra and ESR spectra also confirmed that H bonds, charge-transfer, and covalent bonds were the main adsorption mechanisms between DBSA and poplar leaf biochars. The adsorption mainly took place on the aliphatic C-H, C=O, C-O and aromatic C-H groups.

## Figures and Tables

**Table 1:** BET-N_2_ surface area, pore volume, and average pore size of biochars.

Samples	Surface Area (m^2^/g)	Pore Volume (cm^3^/g)	Average Pore Size (nm)
C300	4.52	0.0042	8.33
C700	375.89	0.2302	3.98
P300	1.29	0.0023	12.27
P700	105.88	0.0721	5.53

**Table 2:** Elemental composition (on a dry mass basis) and atomic ratios of biochars.

Samples	C (%)	H (%)	N (%)	O (%)	H/C	O/C	C/N	(O + N)/C	Ash (%)
C300	61.67	5.41	1.88	31.04	1.05	0.43	38.27	0.46	0.44
C700	83.76	1.50	1.76	10.42	0.21	0.11	55.52	0.12	2.56
P300	59.04	5.87	5.33	27.37	1.19	0.40	12.92	0.47	2.39
P700	69.98	2.19	3.75	18.14	0.38	0.22	21.77	0.27	5.94

**Table 3:** Kinetic equation parameters for the adsorption of DBSA on biochars.

Sample	Pseudo-First Order Kinetic *q_t_* = *q_e_*[1 − exp(−*K*_1_*t*)]			Pseudo-Second Order Kinetic 1/*q_t_* = 1/*K*_2_ × *q_e_*^2^ + *t*/*q_e_* = 1/*v*_0_ + *t*/*q_e_*			
	*K*_1_ (h^−1^)	*q_e_* (mg/g)	*R*^2^	*K*_2_ (g/(mg·h))	*q_e_* (mg/g)	*v*_0_ (mg/(g·h))	*R*^2^
C300	0.148 ± 0.001	34.81 ± 0.035	0.975	0.006 ± 0.000	38.17 ± 0.053	8.636 ± 0.054	0.992
C700	1.754 ± 0.012	35.87 ± 0.060	0.996	0.167 ± 0.004	36.21 ± 0.070	219.0 ± 3.931	0.999
P300	0.675 ± 0.016	36.01 ± 0.048	0.967	0.026 ± 0.001	37.94 ± 0.074	37.43 ± 0.900	0.999
P700	1.108 ± 0.021	34.92 ± 0.095	0.989	0.062 ± 0.002	35.88 ± 0.126	79.82 ± 2.537	0.999

*q_e_* is the adsorbed amount at equilibrium, *q_t_* is the amount of DBSA adsorbed at time t, *K*_1_ and *K*_2_ are the pseudo-first order rate constant and pseudo-second order rate constant, *K*_2_*q_e_*^2^ (*v*_0_) is the initial rate [25,26]. Values are mean ± standard errors.

**Table 4:** Parameters for DBSA different adsorption isotherm equations.

Sample	Linear *q_e_* = *K_P_C_e_*		Langmuir *q_e_* = *Kq_max_C_e_*/(1 + *KC_e_*)			Freundlich *q_e_* = *K_F_C_e_* ^n^		
	K*_P_* (L/g)	*R*^2^	*K* (L/g)	*q_max_* (mg/g)	*R*^2^	*K_F_* (mg/g (L/mg) ^n^)	n	*R*^2^
C300	0.431 ± 0.006	0.980	7.667 ± 0.376	74.82 ± 0.371	0.530	2.026 ± 0.036	0.654 ± 0.002	0.914
C700	0.769 ± 0.069	0.588	57.50 ± 19.33	86.22 ± 0.433	0.981	9.427 ± 0.466	0.468 ± 0.013	0.948
P300	0.442 ± 0.007	0.347	46.33 ± 4.779	71.33 ± 1.652	0.983	6.448 ± 0.231	0.489 ± 0.011	0.745
P700	1.199 ± 0.029	0.592	43.50 ± 4.770	155.9 ± 23.37	0.981	5.047 ± 0.456	0.675 ± 0.037	0.954

*q_e_* is the adsorbed amount at equilibrium, *K_P_*, *K*, and *K_F_* are the partition coefficient, adsorption coefficient and Freundlich affinity coefficient, respectively. *C_e_* is the equilibrium solution concentration, *q_max_* is the Langmuir maximum capacity, and n is the Freundlich linearity constant [27]. Values are mean ± standard errors.

**Table 5:** Correlation coefficient of the adsorption rate constants and the structural characteristics of biochars.

**Index**	***K*_2_**	**Surface Area**		**Pore Volume**	**Average Pore Size**		**C**	**H**	**N**	**O**	**C/** **H**	**O/** **C**	**C/** **N**
Surface area	0.99 **												
Pore volume	0.99 **	1.00 **											
Average pore size	−0.75	−0.80		−0.81									
C	0.97 *	0.99 *		0.99 *	−0.89								
H	−0.85	−0.85		−0.87	0.93		−0.92						
N	−0.42	−0.53		−0.53	0.70		−0.57	0.42					
O	−0.96 *	−0.93		−0.94	0.80		−0.95 *	0.95	0.30				
C/H	−0.85	−0.86		−0.87	0.94		−0.93	1.00 **	0.45	0.94			
O/C	−0.94	−0.93		−0.94	0.85		−0.96 *	0.97 *	0.35	1.00 **	0.97 *		
C/N	0.69	0.78		0.77	−0.72		0.77	−0.54	−0.92	−0.53	−0.56	−0.55	
(N + O)/C	−0.95 *	−0.96 *		−0.96 *	0.89		−0.99 *	0.97 *	0.46	0.98 *	0.97 *	0.99 **	−0.65
Ash	0.25	0.18		0.21	−0.37		0.28	−0.60	0.37	−0.52	−0.58	−0.53	−0.35
Aliphatic	−0.81	−0.84		−0.85	0.99 **		−0.92	0.96 *	0.65	0.86	0.97 *	0.90	−0.70
Heteroaliphatic	−0.89	−0.83		−0.84	0.64		−0.84	0.87	0.03	0.96 *	0.86	0.94	−0.30
Acetal	−0.95	−0.90		−0.90	0.59		−0.87	0.80	0.11	0.95	0.79	0.92	−0.43
Aromatic	0.92	0.92		0.93	−0.91		0.96 *	−0.99 **	−0.43	−0.98 *	−0.99 *	−0.99 **	0.60
Carboxyl	0.23	0.13		0.15	−0.15		0.18	−0.46	0.59	−0.47	−0.43	−0.46	−0.49
Carbonyl	0.50	0.59		0.60	−0.88		0.68 *	−0.65	−0.94	−0.47	−0.68	−0.54	0.84
Aliphaticity	−0.92	−0.91		−0.93	0.91		−0.96 *	0.99 *	0.42	0.98	0.99 *	0.99 **	−0.59
Hydrophobic functional group	0.90	0.84		0.85	−0.61		0.83	−0.84	−0.02	−0.96 *	−0.83	−0.93	0.31
Hydrophilic functional group	−0.90	−0.84		−0.85	0.61		−0.83	0.84	0.02	0.96 *	0.83	0.93	−0.31
Organic free radical concentration	0.97 *	0.98 *		0.98 *	−0.71		0.94	−0.74	−0.56	−0.86	−0.75	−0.84	0.82
**Index**	**(N + O)/** **C**	**Ash**	**Aliphatic**	**Hetero** **Aliphatic**	**Acetal %**	**Aromatic**	**Carboxyl**	**Carbonyl**	**Aliphaticity**	**Hydrophobic** **Functional** **Group**		**Hydrophilic** **Functional** **Group**	
Surface area													
Pore volume													
Average pore size													
C													
H													
N													
O													
C/H													
O/C													
C/N													
(N + O)/C													
Ash	−0.44												
Aliphatic	0.93	−0.42											
Heteroaliphatic	0.90	−0.64	0.72										
Acetal	0.89	0.42	0.67	0.97 *									
Aromatic	−1.00 **	0.52	−0.95	−0.90	−0.87								
Carboxyl	−0.34	0.95 *	−0.22	−0.65	−0.47	0.41							
Carbonyl	−0.63	−0.02	0.83	−0.23	−0.22	0.62	−0.29						
Aliphaticity	0.99 **	−0.53	0.95	0.91	0.86	−1.00 **	−0.42	−0.62					
Hydrophobic functional group	−0.89	0.59	−0.69	−1.00 **	−0.98 *	0.89	0.63	0.20	−0.89				
Hydrophilic functional group	0.89	−0.59	0.69	1.00 **	0.98 *	−0.89	−0.63	−0.20	0.89	−1.00 **			
Organic free radical concentration	−0.88	0.00	−0.75	−0.75	−0.86	0.83	−0.02	0.55	−0.82	0.77		−0.77	

Significant level: * *p* = 0.05, ** *p* = 0.01.

**Table 6:** Intensity distribution in the ^13^C nuclear magnetic resonance (NMR) spectra of biochars.

Samples	Aliphatic %	Heteroaliphatic %	Acetal %	Aromatic %	Carboxyl %	Carbonyl %	Aliphaticity %	Hydrophobic Functional Group %	Hydrophilic Functional Group %
	δ0–60	δ60–90	δ90–110	δ110–165	δ165–190	δ190–230			
C300	22.91	23.98	10.18	35.98	4.81	2.13	61.33	69.07	30.93
C300-DBSA	23.75	17.24	8.78	42.22	5.61	2.39	54.10	74.75	25.25
C700	0.10	1.52	4.28	85.83	5.93	2.35	6.45	90.21	9.79
C700-DBSA	4.29	1.12	3.22	82.07	5.74	3.55	9.51	89.58	10.42
P300	36.66	14.41	7.74	34.25	6.30	0.64	63.20	78.65	21.35
P300-DBSA	35.19	12.80	7.32	36.40	7.14	1.14	60.31	78.91	21.09
P700	7.87	6.96	7.07	69.03	7.17	1.90	24.08	83.97	16.03
P700-DBSA	6.29	6.40	7.75	68.80	7.37	3.38	22.90	82.84	17.16

**Table 7:** Electron spin resonance (ESR) parameters for biochars and its products of interaction with DBSA.

Samples	Organic Free Radical Concentration (Spins/g × 10^14^)	Line Width (Gauss)
C300	0.244	4.7
C300-DBSA	0.297	4.7
C700	11.89	5.3
C700-DBSA	9.632	4.4
P300	0.132	5.6
P300-DBSA	0.196	5.3
P700	1.266	3.2
P700-DBSA	2.599	4.7

Note: The spectroscopic splitting factor is 2.004.

## References

[1] Westall J.C., Chen H., Zhang W.J., Brownawell B.J. (1999). Sorption of linear alkylbenzenesulfonates on sediment materials. Environ. Sci. Technol..

[2] Tan X.L., Fang M., Chen C.L., Yu S.M., Wang X.K. (2008). Counterion effects of nickel and sodium dodecylbenzene sulfonate adsorption to multiwalled carbon nanotubes in aqueous solution. Carbon.

[3] Fernández-Ramos C., Ballesteros O., Zafra-Gómez A., Blanc-García R., Navalón A., Crovetto S.I., Oliver-Rodríguez B., García-Delgado R.A., Vílchez J.L. (2014). Sorption and desorption of alcohol sulfate surfactants in an agricultural soil. Environ. Toxicol. Chem..

[4] Thangalazhy-Gopakumar S., Adhikari S., Ravindran H., Gupta R.B., Fasina O., Tu M., Fernando S.D. (2010). Physiochemical properties of bio-oil produced at various temperatures from pine wood using an auger reactor. Bioresour. Technol..

[5] Dong X.L., Ma L.Q., Li Y.C. (2011). Characteristics and mechanisms of hexavalent chromium removal by biochar from sugar beet tailing. J. Hazard. Mater..

[6] Novak J.M., Busscher W.J., Laird D.L., Ahmedna M., Watts D.W., Niandou M.A.S. (2009). Impact of biochar amendment on fertility of a southeastern coastal plain soil. Soil Sci..

[7] Peterson S.C., Jackson M.A. (2014). Simplifying pyrolysis: Using gasification to produce corn stover and wheat straw biochar for sorptive and horticultural media. Ind. Crops Prod..

[8] Srinivasan P., Sarmah A.K. (2015). Characterisation of agricultural waste-derived biochars and their sorption potential for sulfamethoxazole in pasture soil: A spectroscopic investigation. Sci. Total Environ..

[9] Chen B.L., Chen Z.M. (2009). Sorption of naphthalene and 1-naphthol by biochars of orange peels with different pyrolytic temperatures. Chemosphere.

[10] Zhang J., Liu J., Liu R.L. (2015). Effects of pyrolysis temperature and heating time on biochar obtained from the pyrolysis of straw and lignosulfonate. Bioresour. Technol..

[11] Zhang P., Sun H.W., Li Y., Sun T.H. (2013). Adsorption and catalytic hydrolysis of carbaryl and atrazine on pig manure-derived biochars: Impact of structural properties of biochars. J. Hazard. Mater..

[12] Yao H., Lu J., Wu J., Lu Z.Y., Wilson P.C., Shen Y. (2013). Adsorption of fluoroquinolone antibiotics by wastewater sludge biochar: Role of the sludge source. Water Air Soil Pollut..

[13] Rodríguez-Cruz M.S., Sanchez-Martin M.J., Sanchez-Camazano M. (2005). A comparative study of adsorption of an anionic and a non-ionic surfactant by soils based on physicochemical and mineralogical properties of soils. Chemosphere.

[14] Rodríguez-Sarmiento D.C., Pinzón-Bello J.A. (2001). Adsorption of sodium dodecylbenzene sulfonate on organophilic bentonites. Appl. Clay Sci..

[15] Cotoruelo L.M., Marqués M.D., Rodríguez-Mirasol J., Rodríguez J.J., Cordero T. (2009). Lignin-based activated carbons for adsorption of sodium dodecylbenzene sulfonate: Equilibrium and kinetic studies. J. Colloid Interface Sci..

[16] Sastry N.Y., Séquaris J.M., Schwuger M.J. (1995). Adsorption of polyacrylic acid and sodium dodecylbenzenesulfonate on kaolinite. J. Colloid Interface Sci..

[17] Zhao N., Lv Y.Z., Song G.X., Zhang J. (2016). Sorption behavior of dodecylbenzene sulfonic acid on humic acids from Mollisol and Alluvial soils. Environ. Earth Sci..

[18] Ando N., Kuwabara Y., Kodama T., Mori Y.H. (2012). Surface tensions of aqueous solutions of lithium dodecyl sulfate, sodium oleate, and dodecylbenzene sulfonic acid in contact with methane under hydrate-forming conditions. Fluid Phase Equilib..

[19] Jiang J.H., Zhang L., Wang X.Y., Holm N., Rajagopalan K., Chen F.L., Ma S.G. (2013). Highly ordered macroporous woody biochar with ultra-high carbon content as supercapacitor electrodes. Electrochim. Acta.

[20] Zhao N., Lv Y.Z., Yang X.X. (2017). A new 3D conceptual structures modeling of biochars by molecular mechanics and molecular dynamic simulation. J. Soil Sediments.

[21] Keiluweit M., Nico P.S., Johnson M.G., Kleber M. (2010). Dynamic molecular structure of plant biomass-derived black carbon (biochar). Environ. Sci. Technol..

[22] Chen X.C., Chen G.C., Chen L.G., Chen Y.X., Lehmann J., McBride M.B., Hay A.G. (2011). Adsorption of copper and zinc by biochars produced from pyrolysis of hardwood and corn straw in aqueous solution. Bioresour. Technol..

[23] Chen B.L., Johnson E.J., Chefetz B. (2005). Sorption of polar and nonpolar aromatic organic contaminants by plant cuticular materials: The role of polarity and accessibility. Environ. Sci. Technol..

[24] Li J., Liang N., Jin X., Zhou D., Li H., Wu M., Pan B. (2017). The role of ash content on bisphenol a sorption to biochars derived from different agricultural wastes. Chemosphere.

[25] Rapti S., Pournara A., Sarma D., Papadas I.T., Armatas G.S., Tsipis A.C., Lazarides T., Kanatzidis M.G., Manos M.J. (2016). Selective capture of hexavalent chromium from an anion-exchange column of metal organic resin-alginic acid composite. Chem. Sci..

[26] Wang M.S., Liao L.B., Zhang X.L., Li Z.H. (2012). Adsorption of low concentration humic acid from water by palygorskite. Appl. Clay Sci..

[27] Inyang M., Gao B., Zimmerman A., Zhou Y.M., Cao X.D. (2015). Sorption and cosorption of lead and sulfapyridine on carbon nanotube-modified biochars. Environ. Sci. Pollut. Res..

[28] Arampatzidou A.C., Deliyanni E.A. (2016). Comparison of activation media and pyrolysis temperature for activated carbons development by pyrolysis of potato peels for effective adsorption of endocrine disruptor bisphenol-A. J. Colloid Interface Sci..

[29] Chao H.P., Lee C.K., Juang L.C., Han Y.L. (2013). Sorption of organic compounds, oxyanions, and heavy metal ions on surfactant modified titanate nanotubes. Ind. Eng. Chem. Res..

[30] Yan Q.G., Wan C.X., Liu J., Gao J.S., Yu F., Zhang J.L., Cai Z.Y. (2013). Iron nanoparticles in situ encapsulated in biochar-based carbon as an effective catalyst for the conversion of biomass-derived syngas to liquid hydrocarbons. Green Chem..

[31] Kapoor A., Viraraghavan T. (1997). Heavy metal biosorption sites in Aspergillus niger. Bioresour. Technol..

[32] D’Orazio V., Loffredo E., Brunetti G., Senesi N. (1999). Triallate adsorption onto humic acids of different origin and nature. Chemosphere.

[33] Russell L., Stokes A.R., Macdonald H., Muscolo A., Nardi S. (2006). Stomatal responses to humic substances and auxin are sensitive to inhibitors of phospholipase A_2_. Plant Soil.

[34] Hossain M.K., Strezov V., Chan K.Y., Ziolkowski A., Nelson P.F. (2011). Influence of pyrolysis temperature on production and nutrient properties of wastewater sludge biochar. J. Environ. Manag..

[35] Baes A.U., Bloom P.R. (1989). Diffuse reflectance and transmission Fourier Transform Infrare (DRIFT) spectroscopy of humic and fulvic acids. Soil Sci. Soc. Am. J..

[36] Zhang J.J., Hu F., Li H.X., Gao Q., Song X.Y., Ke X.K., Wang L.C. (2001). Effects of earthworm activity on humus composition and humic acid characteristics of soil in a maize residue amended rice-wheat rotation agroecosystem. Appl. Soil Ecol..

[37] Senesi N. (1992). Binding mechanisms of pesticides to soil humic substances. Sci. Total Environ..

[38] Fang G., Zhu C., Dionysiou D.D., Gao J., Zhou D. (2015). Mechanism of hydroxyl radical generation from biochar suspensions: Implications to diethyl phthalate degradation. Bioresour. Technol..

[39] Senesi N., Testini C. (1982). Physico-chemical investigations of interaction mechanisms between *s*-triazineherbicides and soil humic acids. Geoderma.

[40] Mattson J.S., Mark H.B., Malbin M.D., Weber W.J., Critten J.C. (1969). Surface chemistry of active carbon: Specific adsorption of phenol. J. Colloid Interface Sci..

